# Removal of a large maxillary sinus mucocele via Le Fort I osteotomy during orthognathic surgery: a case report

**DOI:** 10.1093/jscr/rjag974

**Published:** 2026-10-27

**Authors:** Dilara Boylu, Ömer Faruk Boylu

**Affiliations:** Department of Oral and Maxillofacial Surgery, Faculty of Dentistry, Bolu Abant Izzet Baysal University, Karaköy Neighborhood, Mehmed Şemseddin Street, Building No. 6, Gölköy Campus, 14030 Bolu, Turkey; Department of Oral and Maxillofacial Surgery, Faculty of Dentistry, Bolu Abant Izzet Baysal University, Karaköy Neighborhood, Mehmed Şemseddin Street, Building No. 6, Gölköy Campus, 14030 Bolu, Turkey

**Keywords:** maxillary sinus, mucocele, Le Fort I osteotomy, orthognathic surgery, surgical removal

## Abstract

Maxillary sinus mucoceles are uncommon expansile lesions resulting from impaired sinus drainage and mucus accumulation. We report a 19-year-old male with a large left maxillary sinus mucocele identified during planning for bimaxillary orthognathic surgery. Preoperative cone-beam computed tomography showed complete sinus occupation with extension into the nasal cavity and toward the nasopharynx. During the independently planned Le Fort I osteotomy, maxillary down-fracture provided direct access, allowing intact removal of the well-encapsulated lesion. Histopathological examination confirmed a mucocele. At 2-month follow-up, the patient had no nasal symptoms, and postoperative imaging demonstrated marked sinus re-aeration without radiographic evidence of residual or recurrent disease. When independently indicated, Le Fort I osteotomy may provide effective access for simultaneous management of selected extensive maxillary sinus mucoceles.

## Introduction

Paranasal sinus mucoceles are benign, epithelium-lined expansile lesions caused by impaired sinus drainage and progressive mucus accumulation [1–5]. Maxillary sinus mucoceles are uncommon and may expand beyond the sinus, causing bony remodeling and extension into adjacent structures [2–6]. Although previous sinonasal surgery, chronic inflammatory disease, and facial trauma are recognized predisposing factors, some cases have no identifiable cause [1, 3–5, 7–10]. Transnasal endoscopic marsupialization is preferred for most accessible maxillary sinus mucoceles, whereas open or combined approaches may be required for extensive or inaccessible lesions [1, 4, 5, 8, 11]. Le Fort I osteotomy provides wide exposure of the maxillary sinus, nasal cavity, and nasopharyngeal region and has been used to access various midfacial lesions [12–14]. We report the removal of an extensive maxillary sinus mucocele through maxillary down-fracture during independently planned Le Fort I orthognathic surgery.

## Case report

A 19-year-old systemically healthy male was referred for correction of a skeletal Class III dentofacial deformity and reported left-sided nasal obstruction. He had a history of early childhood nasal trauma but no previous sinonasal surgery. Preoperative cone-beam computed tomography (CBCT) demonstrated a lesion completely occupying the left maxillary sinus, extending into the nasal cavity and toward the nasopharynx, with medial sinus wall thinning, ipsilateral airway narrowing, and widening of the natural maxillary ostium (Fig. 1A–C). Following otorhinolaryngology consultation, an antrochoanal polyp was considered the leading diagnosis, with a maxillary sinus mucocele included in the differential diagnosis. Preoperative nasal endoscopy was not performed.

**Figure 1:**
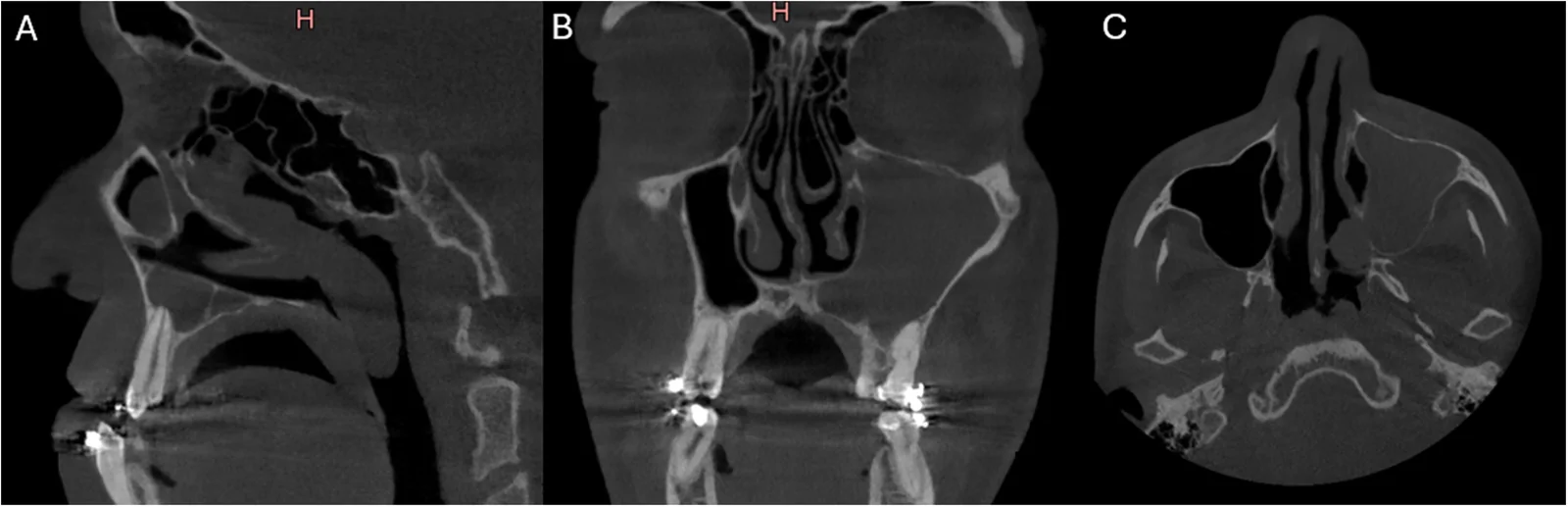
Representative preoperative CBCT sections. (A) Sagittal view demonstrating posterior extension of the lesion toward the nasopharynx. (B) Coronal view showing complete occupation of the left maxillary sinus, thinning and displacement of the medial sinus wall, and narrowing of the ipsilateral nasal airway. (C) Axial view demonstrating complete opacification of the left maxillary sinus, medial extension of the lesion into the nasal cavity, and widening of the natural maxillary ostium.

During the planned bimaxillary procedure, bilateral sagittal split osteotomy was followed by a Le Fort I osteotomy. Maxillary down-fracture provided direct access to a thick-walled, well-encapsulated cystic lesion containing mucoid fluid (Fig. 2A). No purulent material or other evidence of secondary infection was observed; therefore, microbiological culture was not performed. The lesion was separated from the maxillary sinus wall and removed intact (Fig. 2B). Although the natural maxillary ostium appeared widened on preoperative CBCT, its lumen was obstructed by the lesion on direct intraoperative inspection. No antrostomy or surgical enlargement of the natural ostium was performed. The planned orthognathic movements were then completed. Histopathological examination confirmed a maxillary sinus mucocele without evidence of neoplasia.

**Figure 2:**
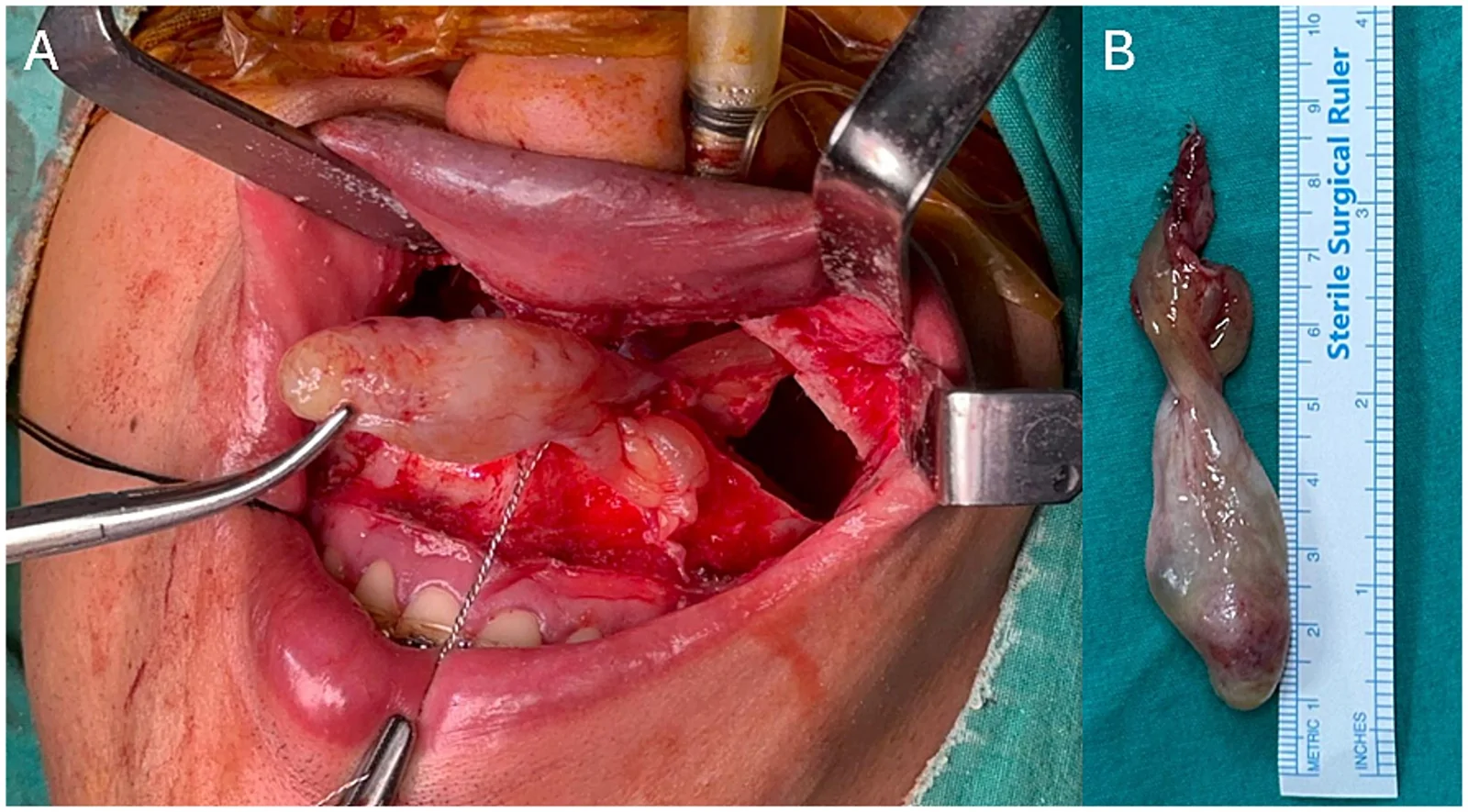
Intraoperative removal and gross appearance of the lesion. (A) Direct visualization of the thick-walled, well-encapsulated cystic lesion within the left maxillary sinus following Le Fort I osteotomy and maxillary down-fracture. (B) Gross appearance of the intact specimen after complete removal, positioned adjacent to a metric ruler.

The postoperative course was uneventful. At the 2-month follow-up, the patient reported no nasal symptoms. Postoperative sagittal, coronal, and axial CBCT demonstrated marked re-aeration of the left maxillary sinus without radiographic evidence of residual or recurrent disease; the natural ostial region appeared radiographically unobstructed (Fig. 3A–C). Preoperative and 2-month postoperative frontal and right-profile photographs documented the facial outcome following orthognathic surgery (Fig. 4A–D).

**Figure 3:**
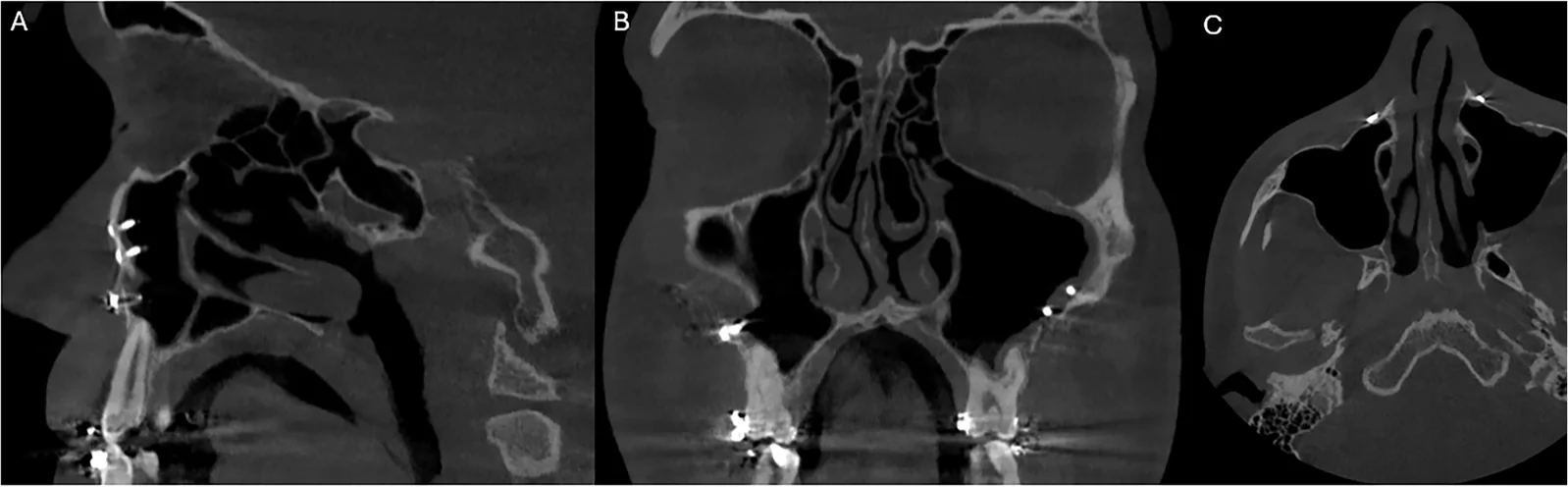
CBCT sections obtained at the 2-month postoperative follow-up. (A) Sagittal, (B) coronal, and (C) axial views demonstrating marked re-aeration of the left maxillary sinus and no radiographic evidence of residual or recurrent disease. The natural maxillary ostial region appeared radiographically unobstructed on the axial view.

**Figure 4:**
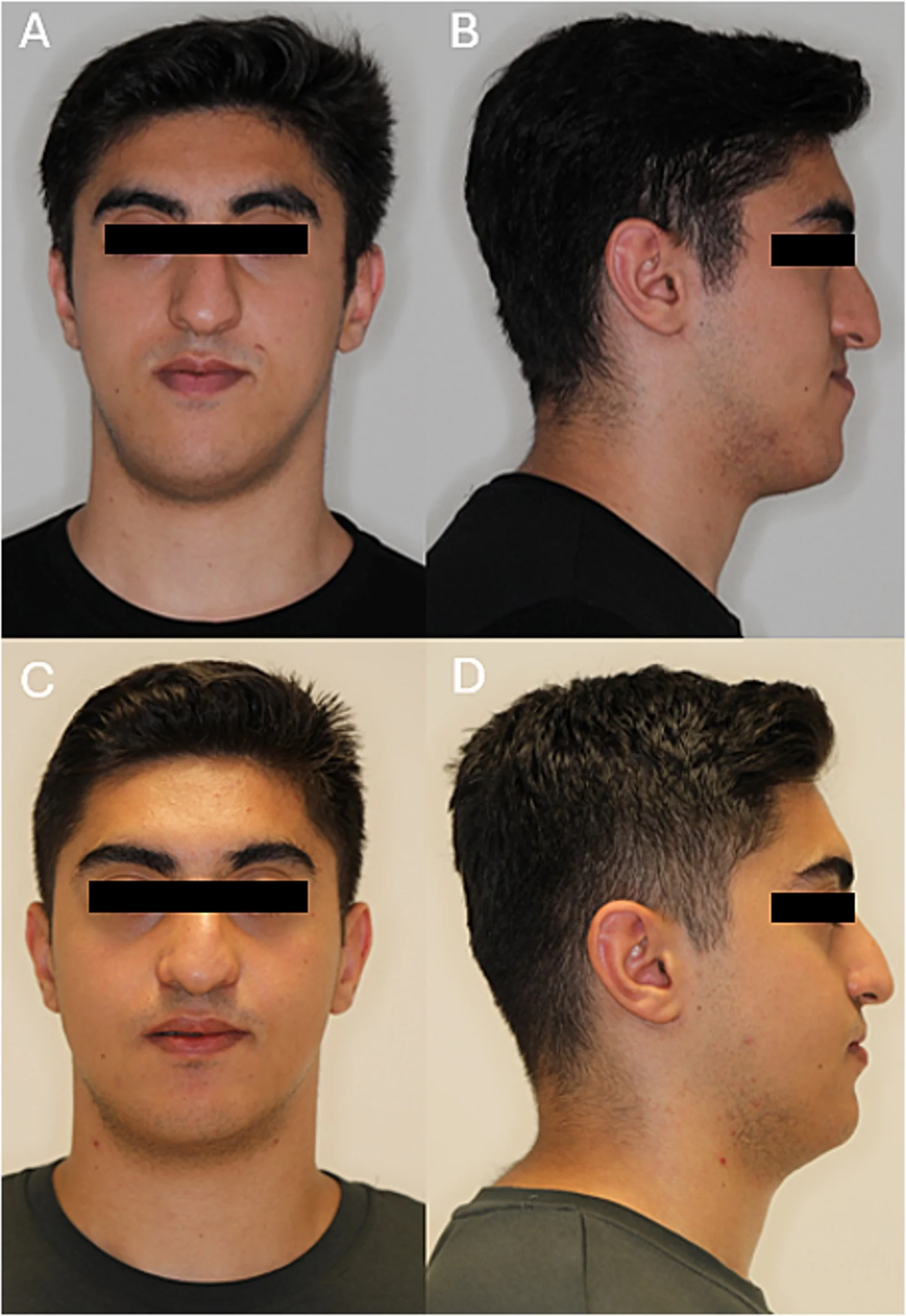
Preoperative and 2-month postoperative facial photographs. (A) Preoperative frontal view. (B) Preoperative right-profile view. (C) Frontal view at the 2-month postoperative follow-up. (D) Right-profile view at the 2-month postoperative follow-up.

## Discussion

Maxillary sinus mucoceles are uncommon, and extensive lesions may cause only nonspecific symptoms [1, 3, 10]. In the present case, the lesion occupied the entire maxillary sinus and extended into the nasal cavity and toward the nasopharynx, yet the only sinonasal symptom was unilateral nasal obstruction. The etiology remained uncertain because the patient had no history of sinonasal surgery, and the relationship between his remote childhood nasal trauma and the mucocele could not be established [1, 3–5, 8–10]. Following otorhinolaryngology consultation, an antrochoanal polyp was initially considered, with mucocele included in the differential diagnosis; histopathological examination established the definitive diagnosis.

Transnasal endoscopic marsupialization is the preferred treatment for most accessible maxillary sinus mucoceles [4, 5, 8, 11]. In this case, however, Le Fort I osteotomy had already been independently planned for orthognathic correction. Maxillary down-fracture provided wide access to the sinus and lesion extensions without requiring an additional surgical approach. En bloc removal was selected because the well-encapsulated lesion could be clearly visualized and separated from the sinus wall without fragmentation. Le Fort I access has similarly been used for other extensive lesions involving the maxillary sinus and adjacent midfacial regions [12–14]. This case-specific use should therefore not be interpreted as an alternative to endoscopic marsupialization for routine treatment.

Although the natural maxillary ostium appeared widened on preoperative CBCT, its lumen was obstructed by the lesion intraoperatively. Neither antrostomy nor surgical enlargement of the ostium was performed. At 2 months, CBCT demonstrated marked sinus re-aeration, a radiographically unobstructed ostial region, and no evidence of residual or recurrent disease. However, these findings do not establish functional ostial patency or durable treatment success, and delayed recurrence remains possible [1, 4, 5, 8, 10, 11].

The principal limitation is the short 2-month follow-up. The absence of preoperative and postoperative nasal endoscopy limited direct endoscopic assessment of the lesion and postoperative functional ostial patency, respectively. Longer clinical and radiographic follow-up, with nasal endoscopy when indicated, is required to assess sustained sinus ventilation and recurrence [2, 5, 6, 10].

## Conclusion

When a Le Fort I osteotomy is independently indicated for correction of a dentofacial deformity, maxillary down-fracture may provide direct access for simultaneous management of an extensive maxillary sinus lesion. In this patient, it permitted intact removal of the mucocele without an additional surgical approach. This case-specific use does not replace endoscopic marsupialization as the standard treatment for accessible maxillary sinus mucoceles [4, 5, 8, 11] but may represent a practical option in selected patients when the planned Le Fort I osteotomy already provides the required exposure [12–14].

## Data Availability

The data underlying this article are available within the article. Additional patient-level data are not publicly available to protect patient confidentiality.
